# Delivering at home or in a health facility? health-seeking behaviour of women and the role of traditional birth attendants in Tanzania

**DOI:** 10.1186/1471-2393-13-55

**Published:** 2013-02-28

**Authors:** Constanze Pfeiffer, Rosemarie Mwaipopo

**Affiliations:** 1 Swiss Tropical and Public Health Institute, Basel, Switzerland; 2 University of Basel, Basel, Switzerland; 3 Department of Sociology and Anthropology, University of Dar es Salaam, Dar es Salaam, Tanzania; 4 Gender Centre, University of Dar es Salaam, Dar es Salaam, Tanzania

**Keywords:** Maternal health, Traditional birth attendants, Health facility delivery, Pregnancy, Tanzania

## Abstract

**Background:**

Traditional birth attendants retain an important role in reproductive and maternal health in Tanzania. The Tanzanian Government promotes TBAs in order to provide maternal and neonatal health counselling and initiating timely referral, however, their role officially does not include delivery attendance. Yet, experience illustrates that most TBAs still often handle complicated deliveries. Therefore, the objectives of this research were to describe (1) women’s health-seeking behaviour and experiences regarding their use of antenatal (ANC) and postnatal care (PNC); (2) their rationale behind the choice of place and delivery; and to learn (3) about the use of traditional practices and resources applied by traditional birth attendants (TBAs) and how they can be linked to the bio-medical health system.

**Methods:**

Qualitative and quantitative interviews were conducted with over 270 individuals in Masasi District, Mtwara Region and Ilala Municipality, Dar es Salaam, Tanzania.

**Results:**

The results from the urban site show that significant achievements have been made in terms of promoting pregnancy- and delivery-related services through skilled health workers. Pregnant women have a high level of awareness and clearly prefer to deliver at a health facility. The scenario is different in the rural site (Masasi District), where an adequately trained health workforce and well-equipped health facilities are not yet a reality, resulting in home deliveries with the assistance of either a TBA or a relative.

**Conclusions:**

Instead of focusing on the traditional sector, it is argued that more attention should be paid towards (1) improving access to as well as strengthening the health system to guarantee delivery by skilled health personnel; and (2) bridging the gaps between communities and the formal health sector through community-based counselling and health education, which is provided by well-trained and supervised village health workers who inform villagers about promotive and preventive health services, including maternal and neonatal health.

## Background

Despite several decades of global health initiatives focused on maternal health, maternal mortality has proven to be an intractable problem. One of the Millennium Development Goal (MDG) indicators for maternal health, the maternal mortality ratio, showed promising signs of a decline in some countries. However, every day 800 women still die during pregnancy and childbirth, and many countries, especially in sub-Saharan Africa, will not meet the MDG target of reducing maternal deaths by 75 per cent from 1990 to 2015 [1].

Attended childbirth is a key component of most developing countries’ primary care strategies and a core part of the essential package of health services. Moreover, regular antenatal care is important to identify women who are at an increased risk of adverse pregnancy outcomes, to ensure birth planning, and to establish good relations between the women and their health care providers. Most maternal deaths occur during labour, delivery or the first 24 hours postpartum. Generally, most intrapartum complications cannot be reliably predicted or prevented, though most of them can be successfully treated with prompt diagnosis and care. Consequently, the role of timely, efficient and appropriate health services cannot be underestimated. Nevertheless, facility delivery rates in sub-Saharan Africa are still some of the lowest in the world [2-7].

Tanzania has made impressive progress in the reduction of child mortality; however, maternal mortality changed insufficiently between 2000 and 2010, and the attainment of the Millennium Development Goal (MDG) no. 5 – Improved Maternal Health – is in jeopardy [8]. The most recent estimate (2010) of Tanzania’s maternal mortality ratio is 454 per 100,000 live births [9]. In 2010, it was estimated that 43% of all women made four or more antenatal care ANC visits. This is a severe decline compared to 2005, when 62% of all women did so. The health facility delivery rates are still rather low in Tanzania. In 2010, 48% of all pregnant women gave birth at home, and 50% delivered in a health facility [9]. The number slightly increased from 2005, when 47% delivered in a health facility [10]. In 2010, 82% turned to a health facility for delivery in urban areas, while in rural areas, only 42% of pregnant women reported delivering their last child in a health facility. 51% of births were assisted to by health professionals, 29% by relatives, 15% by traditional birth attendants (TBAs), and 3% were conducted without any assistance.

Emergency obstetric care services are crucial for handling complicated deliveries. However, in 2010 only 5% of all babies were delivered by caesarean section [9]. This is partially due to delays in timely referral, lack of skilled attendance and functioning blood banks at most hospitals and health centres. About 65% of public hospitals provide Comprehensive Emergency Obstetric Care, whereas only 6% of public health centers provide Basic Emergency Obstetric Care [11]. Furthermore, the referral system faces serious challenges such as limited numbers of ambulances; unreliable logistics and communication systems; and inadequate community-based, facilitated referral systems. Postnatal care is an important component of good maternal and baby health care; however, proper service delivery by the health system and utilization of these services by women in Tanzania remains a challenge. According to the Tanzania Demographic and Health Survey 2010, 65% of women did not receive a postnatal check up at a health facility, and only 31% were examined within two days of giving birth [9].

Multiple factors influence women’s access to skilled health care, including the need to ask permission from their husband or another family member (39%), not wanting to go alone (66%), needing money for treatment (80%), and living a long distance from a health facility (76%) [10]. Moreover, the latter often lack well-trained and motivated staff, the supply of drugs is inconsistent, and their quality of equipment is usually poor [4,12-16].

Therefore, the utilization of traditional birth attendants (TBAs) remains widespread in Tanzania, particularly in rural areas. The Government of Tanzania has officially recognized the potential contribution of TBAs to communities. Strictly spoken, the term TBA only refers to traditional, independent (of the health system), informally trained and community-based providers of care during pregnancy, childbirth and the postnatal period [17]. Several capacity-building programmes, such as the Integrated Management of Childhood Illness (IMCI), have been initiated, and in some cases TBAs are used as community-based counsellors as well as being responsible for monitoring mother and child complications during and after delivery for possible referral [12,13].

However, while the Tanzanian Government promotes TBAs in terms of providing maternal and neonatal health counselling and initiating timely referral, their role does not include delivery attendance. The National Road Map Strategic Plan to Accelerate Reduction in Maternal, Neonatal and Child Deaths in Tanzania, published by the Ministry of Health and Social Welfare, advocates health facility delivery by skilled health workers [11]. This is in line with the recommendations of the World Health Organization (WHO), which states that TBAs can become an important element in a country’s safe motherhood strategy and can serve as key partners for increasing the number of births with a skilled attendant. According to the WHO, TBAs serve as advocates for skilled care, encouraging women to seek assistance from skilled attendants, i.e. health professionals — such as a midwife, doctor or nurse — who have been educated and trained to proficiency in the skills needed to manage normal (i.e. uncomplicated) pregnancies, childbirth and the immediate postnatal period, and in the identification, management and referral of complications for women and newborns: “*Lessons learnt to date show that investing in strategies based solely on TBAs has historically caused governments to delay the development and implementation of strategies for ensuring that skilled attendants are available to all women and newborns. To avoid falling into this trap, the decision to incorporate TBAs into the strategy for the provision of skilled care should be an interim step of a longer-term plan for training and providing sufficient skilled attendants.*” [17: page 8]

Yet, experience illustrates that most TBAs lack sound bio-medical skills and resources, while often still handling complicated deliveries. The same applies for standards of safety and hygiene procedures, which are often not maintained. This is a particular concern in the era of HIV and AIDS. But being community-based, TBAs command a culturally-assigned level of respect and confidence. They are easily accessible, not only in terms of being in the neighbourhood, but also in terms of costs (payment in kind etc.). In addition, they are often appreciated for their affection and motherly manner. Hence their service is indispensable in many settings as they have the potential to bridge - similarly to other health care providers such as Village Health Workers - the gap between the community and the health system [4,12,13].

However, comprehensive and systematic studies on health-seeking behaviour of pregnant women as well as the role of TBAs in reproductive and maternal health are lacking. Therefore, the intended results of this research were:

1. to describe women’s health-seeking behaviour and experiences regarding their use of antenatal (ANC) and postnatal care (PNC) as well as their rationale behind the choice of place and delivery.

2. to learn about the use of traditional practices and resources applied by traditional birth attendants (TBAs) and how these might be linked to the bio-medical health system.

## Methods

### Study design

Using a mixed-methods approach, quantitative as well as qualitative data were collected during August and November 2010. The study focused on various levels within the society and the health system, involving (1) women who had delivered in the past 2 months, (2) TBAs, and (3) community members.

The women were selected according to the following two criteria: (i) women who had delivered in a health facility, and (ii) women who delivered with the support of a TBA. In order to reduce recall errors as far as possible, women who had delivered within 2 months prior to data collection were interviewed retrospectively in detail as to their health-seeking behaviour, treatment(s) and the rationale behind their decision-making, from the time of pregnancy up to delivery. Data were collected by the authors with the support of several field assistants.

### Study setting

In order to learn about urban–rural similarities and differences, the study was conducted in the catchment area of a referral hospital in Ilala District, Dar es Salaam, as well as several villages in the catchment area of a district referral hospital located in Masasi District, Mtwara Region, which is in Southern Tanzania. Ilala District is one of three districts in Dar es Salaam; others are Temeke to the South and Kinondoni to the North. According to the National Census 2002, Ilala has 634,924 inhabitants and covers an area of 273 km^2^[18]. Ilala is commonly referred to as ‘Downtown Dar’, where much of the commerce, banking, and national offices are located [18].

Masasi District is one of the six districts of Mtwara Region, the southernmost region in Tanzania. Other districts in this region include Tandahimba, Mtwara rural, Mtwara Urban, Newala, and Namutumbo. Masasi, located more than 500 km from Dar es Salaam, is the most highly populated district in the region, with 442,573 inhabitants. Although it is currently attracting migrants from other parts of Tanzania, the Makonde remain the dominant ethnic group in the Mtwara Region. Other groups include the Makua and Yao [18].

At both investigated sites, bio-medical health services are delivered by the public health system, which comprises of a network of dispensaries, health centres and hospitals offering a varying quality of care.

### Sampling and methods

Altogether, 200 women were interviewed using a questionnaire (see Additional files 1 and 2): 100 of them in urban and 100 in rural sites. Women who had delivered at health facilities were traced in the maternity ward of the two hospitals. Thus, women who had delivered in the past 6 hours were contacted using a systematic random sampling approach. They were informed about the objective of the study and asked whether they could be visited at home 2 weeks later. Respondents were living across the two large catchment areas of each site. Women who had delivered with the support of a TBA were traced through local authorities and TBAs, using a purposive sampling approach. Initially, it was planned to select 50 women at each site who had delivered at a health facility, as well as another 50 who delivered with the support of a TBA. However, these numbers needed to be adjusted due to the different proportional distribution of the two groups encountered at the two sites. In Dar es Salaam, only women who had delivered at a health facility could be traced, except for one person who had delivered at home with the support of a TBA. In Masasi District, many more women made use of the services of TBAs. In this sample, 50 women had delivered at a health facility and another 50 had opted for a home delivery and the assistance of a TBA. Using a sequential mixed-methods approach, a random subset was selected, comprising 10 women: 5 in Ilala Municipality and 5 in Masasi District. For this selection, determinants such as age, socio-economic background and ethnicity were taken into consideration. Using qualitative methods, the selected women were interviewed in-depth in order to allow for detailed insights into their reasons for health-seeking strategies, experiences and other influencing factors.

Altogether, 14 TBAs (10 in Masasi District and 4 in Ward Ilala, Dar es Salaam) were traced, using convenience sampling through women who had delivered with their support as well as through health facility workers, who often collaborate with TBAs.

In order to learn about the community perspective, 8 Focus-Group Discussions (4 in Ward Ilala and 4 in Maundo) were implemented. The groups were purposefully sampled and divided by age and sex (1. younger women aged 18–39 yrs; 2. older women aged 40 yrs and above; 3. men). Moreover, one group at each site only involved local authorities.

The quantitative and qualitative sample sizes were selected in order to generate sufficient data, allowing a thick, rich description - thereby increasing descriptive validity and interpretative validity. Both the qualitative and quantitative components of this study yielded data that reached data saturation.

Participant observation and informal conversations were applied with actors on various levels for cross-checking and supported the team during data analysis as well as coding.

All participants were asked written informed consent after having been explained the purpose of the study and informed of their right to withdraw their participation at any time.

In accordance with a government circular letter (ref. no. MPEC/R/10/1, dated 4^th^ July 1980), the Vice-Chancellor of the University of Dar es Salaam (UDSM) was empowered to issue research clearance to the university’s staff on behalf of the government and the Tanzanian Commission for Science and Technology (COSTECH). This study received clearance from UDSM with the following ref. no.: *AB3/3(B)*. In addition, the study was authorized by the district coordinators of Reproductive-and-Child-Health (RCH).

### Data analysis

Qualitative data was recorded, transcribed and translated from Kiswahili into English. It was then analyzed and coded with the assistance of the MAXqda2 qualitative data management package (VERBI Software, Marburg, Germany). Additional data from informal discussions and participant observation helped with the interpretation of these data.

Quantitative data was analysed using PASW Statistics 18 (formerly known as SPSS). Here, data was disaggregated to provide information on health-seeking behaviour according to location, age, ethnicity, socio-economic status, etc.

## Results and discussion

### Women’s health-seeking behaviour and experiences at the urban site

In Dar es Salaam, only one woman who had delivered at home could be traced through a TBA and participated in an in-depth interview. The low number of home deliveries is also reflected in the health facility delivery rates, which indicate that in urban areas in Tanzania, more than 82% of women turned to the health system for delivery in 2010 [9]. In Dar es Salaam, the biggest city, the numbers are probably even higher. Findings from FGDs, in-depth and structured interviews revealed that deliveries with the assistance of a TBA are not a common practice in Ilala; instead, women prefer using the services of health facilities. All respondents mentioned a preference for delivering at a health facility. TBAs were blamed for being the main cause of maternal and neonatal deaths. In addition, none of the interview partners reportedly knew of any practising TBA in Dar es Salaam.

The majority of the respondents interviewed with a questionnaire (n = 100) were married (56%), already had more than 2 children (45%) and reported ‘trade/business’ (49%) as their main source of income. More than 60% had a primary and less than 30% a secondary education background. Asked about their reasons for delivering at a health facility, ‘good service’ (48%) and ‘security’ (34%) were most frequently mentioned.

“*What makes me prefer going to the hospital is to avoid problems. In case they occur while I am at the hospital, they [health workers] will know what to do.*

*But if you are at home and some complications occur it will take time to reach a hospital in order to save you.*” (In-depth interview, Dar es Salaam)

None of the women interviewed either the qualitative or quantitative interviews (n = 105) made use of the services of a TBA during and after pregnancy. The only woman who had delivered with the assistance of a TBA reported realizing her labour pains too late and therefore not having enough time to go to the nearest hospital. She was assisted by her mother-in-law, who happened to be a TBA. After delivery, she was immediately referred to the nearest hospital and went there for follow-up treatment. Asked about the delivery practices, she said:

*“[The TBA used] cotton, gloves, razor blade, Dettol [an Antiseptic] and a bottle with a medicine, in case after delivery you bleed too much, they will inject you to stop the bleeding, even now when the TBA comes to assist you she will ask you, do you have utensils? Because now-a-days they also use modern utensils not like in the past where they were not using these things.”* (In-depth interview, Dar es Salaam)

46% of respondents reported that the place of delivery was their personal choice. About 20% were persuaded by health staff whom they regarded as trustworthy and knowledgeable. Family members (6%) and husbands/partners (9%) did not seem to play an important role. These results were confirmed in the qualitative interviews.

On average, the women spent 5 to 6 USD for delivery-related treatment and tools that were mainly for the compulsory delivery kit. Costs for kits vary depending on the dispensary and the type of packaging (set or individual items). None of the women complained about having to bribe health workers; in contrast, many appreciated the fact that the treatment was free of charge.

The majority of all interviewed women paid between 6 and 10 USD for transport. Most of the respondents travelled by taxi, 6 women used their own car and another 6 reached the hospital by bus. 38% of the respondents had to travel for less than half an hour, while 39% needed 30 minutes to one hour to reach the hospital. 18% had a longer distance to overcome and travelled for more than one hour. Nevertheless, transport did not constitute an access barrier due to a relatively good, reliable and affordable public transport system.

More than 90% of all women were received well within 30 minutes after arrival at the hospital. However, some who needed further examinations complained about delays.

“*When I reached there, I found a nurse who asked me how I felt, whether I feel the baby will come soon, and I said, yes, then she told me to take off my clothes and she checked my vagina to see if the baby was near and after that she told me to go and lay on the bed and wait.*” (In-depth interview, Dar es Salaam)

More than 55% of respondents evaluated the delivery treatment as ‘very good’, while 39% rated it to have been ‘good’. However, the selected referral hospital is generally well-known for its good service. Not all hospitals in Dar es Salaam were rated as positively and results can thus not be generalised.

99% of respondents reported more than 2 antenatal care (ANC) visits during their pregnancy, which is significantly higher than the country’s average. According to the Tanzanian Demographic and Health Survey 2004–2005, 96% of all pregnant women attended antenatal care at least once [10]. However, the high attendance rate found in this study does not provide any information about the quality of the antenatal care services.

40% of patients developed health problems themselves and/or their neonates fell sick. 34% decided to turn to a health facility for treatment, while 6 women opted for self-treatment.

### Women’s health-seeking behaviour and experiences at the rural site

The majority of the women (49%) interviewed with a questionnaire in Masasi District were married, had more than 2 children (39%), stated farming as their main source of income (67%) and a primary school education background (65%) (n = 50 health facility delivery; n = 50 home/TBA delivery). The level of education seems to influence their place of preference for delivery: 11% of the women utilizing services of TBAs, as compared to 4% of women who had turned to health facilities, had no formal education. Younger women aged 15 to 22 delivered more often with the assistance of a TBA than women 23 years and older.

Qualitative data revealed that the primary factor influencing women’s place of choice for delivery was ‘convenience’. The location of and distance to a single health facility supporting a catchment area of up to 7 villages as well as the poor transport system were push factors towards TBAs and home deliveries. In addition, the low rate of fatalities associated with home deliveries gave women a certain degree of confidence that they were safe. The saying “*But I delivered the first baby safely at home! [mbona mtoto wa kwanza nimezaa nyumbani salama!]”* reflects the belief that it is safe and therefore culturally acceptable to deliver at home.

Respondents to the questionnaire who had delivered at a health facility mentioned ‘professional service’ (51%) as their main reason to turn to the formal health system, while women who had given birth with the support of a TBA had various motivations. ‘Unexpected delivery’ (52%) and ‘realizing it too late’ (28%) were mentioned most frequently as preventing them from travelling to the nearest facility. A woman in Masasi District summarised it as follows: “*The point of delivery comes suddenly like rain [uzazi wenyewe unakuja ghafla, kama mvua].”* In addition, a shortage of skilled personnel makes women seek the assistance of a TBA, who is both accessible and available.

The qualitative interviews revealed that many women prefer delivering in a private and confidential environment with the assistance of someone from within their community, someone they trust and know well. Although they have been advised during MCH visits to avoid doing so, they often prefer using the quick services of a community-based TBA.

This is confirmed by other studies in Mtwara Region [12,13]. Some women, who prefer hiding their pregnancy due to cultural reasons, shunned health facilities as public spaces that lack privacy and are not conducive. A woman in Masasi District confirmed this when she said:

*“Local women have a custom of remaining at home [when labour pains begin]. You can not go to the dispensary too early and wait there for everyone to see you.”* (In-depth interview, Masasi District)

Another study in Mtwara Region confirmed that younger women often do not want to deliver in the presence of male health workers [12]. While 37% of women with health facility delivery decided on their own that they wanted to seek support from the health system, 23% were influenced by health workers, who seemed to play an important role in decision-making, and 12% by their partners. Fewer respondents mentioned influence by relatives (5%) and TBAs (2%). Interestingly, the majority of TBA users (62%) reported deciding on their own where to deliver, which might be due to their lack of time to consult others because of ‘unexpected delivery’ or realizing delivery ‘too late’. 18% of respondents, especially adolescents, mentioned the influence of relatives. Keeping in mind that in the rural research site, a higher number of women below 20 years of age turned to a TBA than to a health facility, it can be argued that they were influenced by elders, who might favour traditional practices. Additionally, teenagers often depend on their relatives due to lack of financial resources.

Health facility deliveries were free of charge; however, this did exclude delivery kits, which cost on average of 3 USD. Several women complained about difficulties in terms of (1) mobilizing resources for the kits and (2) accessing delivery kits in their villages respectively. Often, kits were not available at community level. Attendance by a TBA was generally free of charge. They were only paid a token amount, usually about 1–1.50 USD. The use of delivery kits was demanded by some TBAs. Evidence from several studies emphasizes that the cost of accessing care is a critical determinant [15,16]. Despite the fact that Tanzania has a high coverage of health facilities as compared to neighbouring countries, delivery at health facilities often involved having to travel long distances. Bad road conditions, coupled with inadequate and unaffordable transport, often make it impossible for the poor to reach the facilities [15]. In the female’s FGD at the rural site, women claimed that they had to pay between 2 and 6 USD for bicycle hire to the nearest dispensary, depending on the time at hand as well as the owner of the bicycle. Hardly any travel costs had to be paid by TBA users since almost all of them were living nearby.

Women differentiated between the desire to consult professional services that could be received in certain health facilities and the costs related to these services, which often seemed to be a challenge. During the FGD in Masasi District, one woman explained it as follows:

“Everyone makes independent decisions about where to go when one has a health problem. When we have malaria we decide to go to a health facility which is 4 km away because of their equipment. It is a Mission run facility and we have to pay. The government dispensary is 7kms away but they do not have the needed equipment, you are only given medicine. But for pregnancy and related issues such as labour we go to the government dispensary, because the service is free.”

The waiting time for attendants at a health facility mainly ranged from below 30 minutes (32%) up to more than 30 minutes (33%); however, 7% of the women complained about having had to wait for more than 2 hours. On the other hand, the waiting time for TBA services was minimal. The treatment of patients by both TBAs and health facilities was rated as ‘good’. 99% of all respondents delivering at a health facility made more than 2 ANC visits during their pregnancy. Also, the majority (86%) of all women who decided in favour of a TBA delivery made at least 2 visits to a health facility, which is significantly higher than the average rates of ANC use in the country. Despite these high rates, delivery at health facilities still remains low. Several studies show that this can be attributed to the above-mentioned access factors such as too long distances to still make it in time to the nearest facility and scarce public and private transport [12,13,15].

None of the women who delivered at a health facility said that they used a TBA during pregnancy. However, there is a tendency among women who delivered with the support of a TBA to use ANC services less often than their counterparts who delivered at a health facility.

It was not common to use services of TBAs during pregnancy. Women with health facility deliveries reportedly never visited them, while only 7 out of 50 women with TBA delivery consulted TBAs before delivery. The latter received periodic consultations by TBAs, starting from the 7^th^ month of pregnancy.

In case of health problems in the first week after delivery, the majority (55%) of the women who delivered with a TBA made use of the formal health system: “*we seek [professional] examination [tunafuata vipimo]*”. However, a significant amount (36%) also turned to a TBA for advice.

Asked about the reasons why some women did not want to deliver with the assistance of a TBA, women who did so mainly mentioned ‘poor services’. This stands in contrast to the reasons given by women who delivered at a health facility and mentioned the ‘good services’ there. It illustrates dissatisfaction with TBAs in terms of skills and equipment. Despite the low quality of care encountered in many, especially rural health facilities, women still have more trust in their services [12,14]. Although they would have liked to deliver at a health facility, access barriers often prevented them from doing so. Despite the community members’ dissatisfaction with TBAs, several studies highlight the role of them as service providers and argue in favour of an integration of TBAs into the formal health system [19-21].

### Practices and resources applied by urban TBAs

It was very difficult to trace TBAs in Dar es Salaam. Various networks in Ilala Ward were used in order to identify them; however, only 4 TBAs could be found. All of them were female and aged above 60 years. They reportedly practiced only in cases of emergency and preferred women to go to a health facility for delivery. In the previous two years, they had only assisted very few women, mainly family members and close neighbours.

*“It depends […] you help her at that moment and other pregnant women turn to the hospital, it might be twice in a year.”* (In-depth interview, Dar es Salaam)

However, they were afraid of complications and being held responsible for maternal and neonatal deaths.

*“When we see there is a problem, we rush the pregnant woman to the hospital, we help someone who does not have any complications, when you look at someone, and you see she has problems you must rush her to the hospital, because even at the hospital, they tell you to help someone, who does not have any complications, but the one with problems should be rushed to the hospital.”* (In-depth interview, Dar es Salaam)

They did not provide any specific ANC- or PNC-related services or traditional practices, but offered general advice if needed. Moreover, no traditional medicine was prepared or prescribed by them. They regarded their role as simply assisting during delivery and afterwards encouraging immediate referral to the health facility. All of them highlighted the importance of delivery kits. If none had been bought by their patients, the TBAs used their own kits comprising of gloves, disinfectant, sterile razor blade, thread and cotton.

TBAs reportedly did not use any traditional tools or medicine during delivery. In general, their knowledge related to hygiene standards and risk signs, and the importance of referral was surprisingly high. One out of 4 TBAs had attended several trainings (on HIV/AIDS, maternal and neonatal health, safe delivery etc.) by health workers in the past and used her knowledge to inform colleagues and the community. All of them felt well-accepted and supported by health workers and regarded them as very skilled partners and key sources of information.

It can be concluded that the interviewed TBAs in Ilala District regarded themselves as community-based providers of care who mainly assist during delivery, especially in cases of emergency. The vast majority were elderly and had learnt their skills through relatives or observations made during deliveries. Their numbers are declining, and new recruits are rare. Several of them had received training on maternal and neonatal health (danger signs, clean delivery standards, HIV/AIDS etc.) by non-governmental as well as governmental organizations and collaborated with the health system, for instance by referring women who had delivered with their assistance and their neonates for follow-up treatment to hospitals. These days, TBAs in Ilala District are characterized by a biomedical orientation.

### Practices and resources applied by rural TBAs

In Masasi District, interviewed TBAs were usually 50 years or older. Most of them enlisted in the 1980s after a government directive to Village Governments called on to do so. Their numbers have dwindled since formal recruitment by Village Governments stopped, although new recruits who were assisting older TBAs have been enlisted in a few villages. The decreasing number of formally recognised TBAs has rarely been an issue in the communities. This is due to local practices in several places within Masasi District, where childbirth is traditionally regarded as a family affair. Birth attendants are selected amongst the women of the family; therefore, home deliveries by selected female family members are still very common. In 2005, out of the 53% of births in Tanzania which took place at home, 31% were assisted to by relatives, 19% by traditional birth attendants (TBAs) and 3% were conducted without any assistance [10]. In a household survey conducted in Mtwara in 2007, 43% of women delivered in a facility, 30% by a TBA, 22% by others such as relatives, and 3% had no assistance whatsoever [12,13].

It is thus evident that TBAs and relatives who assisted with home deliveries represent a crucial link between communities and health facilities, albeit their informal role. This is due to the limitations of the formal health system in rural areas. In this survey, TBAs were seen to be more accessible, which could be explained with the psychosocial care they gave to patients, even though a few respondents branded them as driven by money. However, most of the TBAs in the study confirmed that they were usually paid a meagre 1 USD. TBAs justified their commitment as *“helping each other [kusaidiana wenyewe kwa wenyewe]*” but at the same time regarded it as a service that requires specific expertise.

Special local practices were believed to reduce complications during delivery. According to local tradition, TBAs usually assist from the 7^th^ month of pregnancy and encourage women to “*open up the path [kupanua njia]*” by using a hand to manipulate the uterus.

*“Starting from the 7*^*th*^*month, the TBA or the ‘Somo’ [an older female confidant and advisor, a traditional arrangement in south-eastern Tanzania] comes to teach you [the pregnant woman] how to widen the passage for the baby. For the first time she shows you by putting her hand [in the vagina] and teaching you how to do it, how to perform it. You put in your hand and you circulate it around the area, and keep on doing it until you get close to delivery. Some of the Somo do not use protection, just their hands.”* (In-depth interview with a TBA, Masasi District).

Another application includes the *“mlenda”* leaves, which are very lubricious. When applied on the uterus by hand, the leaves supposedly make it easier for the neonate to move through the birth canal.

*“That is why in the past there were few delivery complications, these days when a women has a few complications she is rushed to get an operation [at a health facility].”* (In-depth interview with a TBA, Masasi District)

In general, TBAs stressed being cautious assisting during deliveries without protection. One of them insisted that they had to be careful these days because of HIV/AIDS. Another one explained that

*“These days we look at the woman’s ANC card and if I see nyota, [a symbol that refers to women needing special attention] I just tell her you have to go to the hospital because she has already been marked as someone needing special assistance during delivery, so what I do is when I see nyota, nyota, nyota – I just tell them go to the hospital, and do not even go to a local dispensary.”* (In-depth interview, Masasi District)

The TBAs’ knowledge about cleanliness and hygiene during delivery was high, particularly because of HIV/AIDS awareness campaigns. One of the TBAs in Masasi District summarised her worries as follows:

“*I was called to assist a young woman at night, but when I asked her about gloves, she said she had none, so I refused to assist her, I told her husband to take her to the health facility […] I did not want to risk touching her without protection. She was also stubborn […] she wouldn’t follow my advice.*”

The practical application of clean delivery practices during home deliveries depended on whether the instruments were available, which was not often the case. TBAs commonly used gloves and often even demanded to use them, as the above experience illustrates. In addition, women reiterated their understanding of getting prepared with a delivery kit in all villages visited.

TBAs regarded themselves as experienced, but not as experts. They had learned their trade through observation. Practices related to attending to the neonate were seen to be common across the villages. Neonates were delicately handled because of the conviction that they just arrived “*from the warmth of the womb to a new environment*”. In some cases, neonates were even believed to need ‘protection’ before being confronted with people. Here, the link between TBAs and traditional healers became obvious. A TBA in Masasi District explained:

“I use mitishamba [herbs] to cleanse the baby, we do not bathe him/her immediately after birth. In the past my grandparents used to give me this powdered mitishamba, I do not know the name. You use this stuff when you cleanse the baby for the first time to protect it from evil hands.”

TBAs at the rural site regarded themselves as community-based providers of care, who mainly assist home deliveries, not necessarily because of emergencies, but also due to a choice by the women themselves. The majority is over 50 years of age and learnt their skills through a form of ‘apprenticeship’ under more experienced birth attendants. Most of them were enlisted in the 1980s following a government directive to Village Governments. Their numbers have dwindled since their formal recruitment by the villages stopped. They combine both traditional as well as biomedical concepts. Several of them have basic knowledge about clean delivery practices and collaborate with the health system. At the same time, traditional and often harmful practices are used to support mother and child. This finding is in line with other studies, indicating that TBAs can contribute to maternal deaths due to lack of knowledge and the use of traditional practices [22].

## Conclusions

The findings show that TBAs at the selected sites did not work independently of the health system. Due to training provided by several non-governmental organizations and the Government in the past decades, many TBAs possess a basic understanding about maternal and neonatal health issues and are influenced by biomedical orientations. This is also reflected in the material they use during delivery (e.g. gloves).

At both sites, the interviewed TBAs were not acting as traditional healers. Although women pointed out that TBAs are often not as skilled as health workers, community members appreciated TBAs as a link between the community and the formal health system, due to their accessibility and availability. Moreover, TBAs are respected for their motherly and caring behaviour. Besides their positive reputation in terms of providing psycho-social support, no positive traditional practices and resources could be identified. In contrast, some of the practices and medicine encountered were possibly harmful and may have put the lives of mother and child at risk. According to the interviewed women, the traditional sector does not offer a promising complement to the formal health system, but is mainly used where skilled services are inaccessible.

The results from the urban site (Ilala District, Dar es Salaam) show that significant achievements have been made in terms of promoting pregnancy- and delivery-related services by skilled health workers. Pregnant women showed a high level of awareness and clearly preferred delivering at a health facility. Only in rare occasions, mainly in cases of emergency, women turned to a TBA for delivery. This is also reflected in the low number of TBAs who could be traced by this study in the city. Access to health care services is easier in urban than rural areas due to the availability and reliability of transport, better transport systems and roads as well as a higher coverage of health facilities. Costs are regarded as low and mainly involve delivery kits plus transport fees. Health workers strive for offering good services in a resource-poor setting. For this study, it needs to be acknowledged that the selected hospital has a better reputation than other urban health facilities, and findings thus cannot be generalized.

Moreover, education levels in Ilala District are higher than at the rural site. Women seemed to be well-informed about the importance of ante- and postnatal care services as well as warning signals during pregnancy. Their main sources of information were primarily health workers and secondly health-related TV and radio campaigns. The scenario is different at the rural site, where an adequately trained health workforce and well-equipped health facilities are not yet a reality. Instead, health facilities are still understaffed and located too far away for many villagers, resulting in home deliveries with the assistance of either a TBA or a relative.

The frequent use of ANC services found at both sites does not translate into a health facility delivery. This is rather due to access factors [23]. The main obstacle preventing women from delivering at health facilities found in this study was ‘realizing labour too late’ to travel the long distances to the nearest one. Scarce public and private transport poses an additional barrier to timely and appropriate treatment. Moreover, lack of money for transport and additional delivery-related costs, such as delivery kits, prevented women from turning to the formal health system. In the light of the above, it becomes clear that the parturient should be exempted from all out-of-pocket payments at the facility level. This needs to include delivery kits, which had to be purchased by the women at both research sites., Lack of confidentiality and rude behaviour by health workers were mentioned as an additional challenge.

While TBAs, who are mainly elderly people, are slowly phasing out, home deliveries are not, nor will they be in the foreseeable future. The role of community-based actors, whether TBAs or relatives, cannot be underestimated. It is argued that the problem of home deliveries will not be solved by focusing mainly on improving the formal health system. Practices of community members cannot simply be changed by facilitating access. A more holistic approach is needed.

On the village level, community-based interventions that sensitize and inform both women and men about maternal and neonatal health risks and services are therefore crucial. While there is no evidence on the contribution of trained TBAs to reducing maternal and neonatal mortality rates, services offered by Village Health Workers have globally contributed to the decline of maternal and child mortality rates [24].

Building on the findings of this study, it is argued that instead of focusing on the traditional sector, more attention should be paid on a systematic, multi-facetted approach to overcome health-system constraints as well as on community-based programmes [25-27]:

(1) Improving access to and a strengthening of the health system (investment in the capacities of health facility staff, especially of female nurses and midwives; overcoming access barriers by exploring a variety of options, including maternity waiting homes as well as transport improvements such as different types of ambulance services and community-based transport schemes, guaranteeing a private and confidential environment and adequacy of staff, providing sufficient equipment and commodities, exploring the potential of free distribution of delivery kits in health facilities); and

(2) Bridging the gaps between communities and the formal health sector through community-based counselling and health education provided by well-trained and supervised village health workers (also incorporating existing TBAs) who are integrated into the formal health system and who inform villagers about promotive and preventive health services in general as well as maternal and neonatal health-related messages in particular [20].

These activities are in line with the Tanzanian Primary Health Services Development Programme (MMAM), which includes plans to establish a cadre of community health workers who receive formal, standardized training and get paid by the system [28].

At the moment, several formative research studies that aim at evaluating the effectiveness and cost of scaleable strategies are being implemented in order to improve neonatal and maternal health in rural southern Tanzania through community-based interventions and by introducing community health workers [personal communication with Dr. Fatuma Manzi and Kate Ramsey].

## Competing interests

The authors declare that they have no competing interests.

## Authors’ contributions

CP and RM were involved in the design and implementation of the study, field work, data management, analysis, interpretation of the data, and writing of the manuscript. All authors have read and approved of the final manuscript.

## Pre-publication history

The pre-publication history for this paper can be accessed here:

http://www.biomedcentral.com/1471-2393/13/55/prepub

## Supplementary Material

Additional file 1 Questionnaire for women who had delivered at a health facility. Click here for file

Additional file 2 Questionnaire for women who had delivered with the assistance of a Traditional Birth Attendant. Click here for file
