# Supplementary material for: Delivering at home or in a health facility? health-seeking behaviour of women and the role of traditional birth attendants in Tanzania
Source: BMC Pregnancy Childbirth. 2013 Feb 28;13:55. doi: 10.1186/1471-2393-13-55 (PMC3599352; doi:10.1186/1471-2393-13-55)
Supplement: Additional file 2 — Questionnaire for women who had delivered with the assistance of a Traditional Birth Attendant. [file 1471-2393-13-55-S2.pdf]

MATERNAL HEALTH AND TRADITIONAL KNOWLEDGE IN TANZANIA

DELIVERY WITH THE SUPPORT OF A TBA

QUESTIONNAIRE

QUESTIONNAIRE NO | | |

REGION | |

VILLAGE/WARD ______________________

DATE OF INTERVIEW

				2	0	1	0

INTERVIEWER:

We are interviewing women, who delivered in the past 6 months, to get a better understanding about their preferences of place of delivery.
Your participation in the study is completely voluntary. We would also like to assure you that all information collected in the course of the study will remain confidential.

→ *Ask for oral consent.*

Thanks a lot for your participation. In case you have any questions, please let us know.
Please ask when you have a problem understanding a question.

DEMOGRAPHICS

No.	Question	Response	Code
1	Age	---------------------	1
2	Marital status	Never married □<br>Married □<br>Divorced/separated □<br>Remarried □<br>Widowed □	1<br>2<br>3<br>4<br>5
3	Number of children	No child □<br>1 Child □<br>2-5 Children □<br>Over 5 children □	1<br>2<br>3<br>4
4	Education	No formal education □<br>Primary education □<br>Secondary Education □<br>University □	1<br>2<br>3<br>4
5	Religion	Muslim □<br>Catholic □<br>Lutheran □<br>Anglican □<br>Protestant □<br>Pentecostal □<br>Charismatic □<br>Seven Days Adventists (SDA) □<br>Jehovah Witness □<br>Traditional □<br>No religion □<br>Other, specify_________________	1<br>2<br>3<br>4<br>5<br>6<br>7<br>8<br>9<br>10<br>11<br>96
6	Ethnic group	Makonde □<br>Yao □<br>Mawia □<br>Makua □<br>Wamwela □<br>Haya □	1<br>2<br>3<br>4<br>5<br>6

		Matumbi □<br>Zaramo □<br>Chaga □<br>Sukuma □<br>Other, specify________________	7<br>8<br>9<br>10<br>96
7	Family's main source of income and livelihood?	Trade/Business □<br>Farming □<br>Fishing □<br>Farming + Fishing □<br>Official employee □<br>Casual labour □<br>Other, specify________________	1<br>2<br>3<br>4<br>5<br>6<br>96

PERSONAL EXPERIENCES AND RATIONALE

1. For the child you just gave birth to, was it your first pregnancy?
Tick the appropriate answer

1. Yes	2. No
☐	☐

2. What were your reasons for delivering with the support of a TBA?

....

....

....

....

3. Who influenced you in deciding where to deliver?
Tick all appropriate answers

1. Own decision	2. Husband/ partner	3. Own family	4. Family of the partner	5. Neigh-bours	6. Local authorities	9. Other, specify
☐	☐	☐	☐	☐	☐	………..............................

4. What were the main benefits you expected to get during treatment?

....

....

....

....

5. How much did the treatment cost?

………

………

6. Did you pay the TBA in cash or kind?

Tick the appropriate answer

1. Cash	2. In kind	3. No payment	9. Other, specify
☐	☐	☐	………..........................

7. How far did you travel for the service?

Tick the appropriate answer

1. No travelling needed	2. Below 30 minutes	3. 30 minutes – 1 hour	4. 1 hour – 2 hours	5. More than 2 hours	9. Other, specify
☐	☐	☐	☐	☐	………..........................

8. How much did you pay for travelling, accommodation or other expenses related to the delivery?

………

………

9. How long did you wait for the service?

Tick the appropriate answer

1. Imme-diately	2. More than 30 minutes	3. Over 1 hour	4. Over 2 hours	5. No service	9. Other, specify
☐	☐	☐	☐	☐	………..........................

10. Were you accompanied?

Tick the appropriate answer

1. Yes	2. No
☐	☐

10. 1 If yes, by whom?

Tick all appropriate answers

1. Husband/ Partner	2. Parents	3. In-laws	4.Other relatives	5. Neigh-bours	9. Other, specify
☐	☐	☐	☐	☐	………..........................

11. How were you treated during delivery?
Tick the appropriate answer

1. Very good ☐	2. Good ☐	3. Not so good ☐	4. Bad ☐	9. Other, specify ……............................

12. What treatments were you given?
....
....
....
....

13. What medicines were you given?
....
....
....
....

14. How many pregnancy related visits did you make during your pregnancy at a medical health facility?
Tick the appropriate answer

1. None ☐	2. 1 ☐	3. 2-3 ☐	4. 4 ☐	5. More than 4 ☐	9. Other, specify ……............................

15. Did you partly or completely comply with the antenatal care services given by the medical health facility during pregnancy?
Tick the appropriate answer

1. No compliance ☐	2. Partly comply ☐	3. Com-pletely ☐	9. Other, specify ……............................

15.1 Why ?
....
....
....
....

16. How many pregnancy related visits did you make at the TBA during your pregnancy?
Tick the appropriate answer

1. None	2. 1	3. 2-3	4. 4	5. More than 4	9. Other, specify
☐	☐	☐	☐	☐	……...........................

17. What did you like about the service?
……
……
……
……

18. What did you dislike about the service?
……
……
……
……

19. Did anything go wrong during delivery?
Tick the appropriate answer

1. Yes	2. No
☐	☐

19.1 If no, please continue with Q. 20; If yes, what?
……
……
……

19. 2 What did the TBA do?
……
……
……
……

19.3 Were you referred to a health facility?
Tick the appropriate answer

1. Yes	2. No
☐	☐

19.4 If no, please continue with Q. 19.6; if yes, was there any assistance by the TBA related to the referral?

………

………

………

………

19.5 How long did it take to access services from a medical health facility?
Tick the appropriate answer

1. Imme-diately	2. More than 30 minutes	3. Over 1 hour	4. Over 2 hours	5. No service	9. Other, specify
☐	☐	☐	☐	☐	………………………………

19.6 What was the outcome?

………

………

………

………

20. In the first week after delivery, have you suffered any complications?
Tick the appropriate answer

1. Yes	2. No
☐	☐

20.1 If no, please continue with Q. 22; If yes, which ones?

………

………

………

………

21. Did you go for treatment to a TBA or to a medical health facility?
Tick the appropriate answer

1. TBA	2. HF	3. Both	9. Other, specify
☐	☐	☐	………........................

21.1 Why?

……

……

……

……

21.2 What was the outcome?

……

……

……

……

22. In the first week after delivery, has your baby suffered any complications?
Tick the appropriate answer

1. Yes	2. No
☐	☐

22. 1 If no, please continue with Q. 24; If yes, which ones?

……

……

……

……

23. Did you go for treatment to a TBA or to a medical health facility?
Tick the appropriate answer

1. TBA	2. HF	3. Both	9. Other, specify
☐	☐	☐	………........................

23.1 Why?

………

………

………

………

23.2 What was the outcome?

………

………

………

………

24. Why do some women not consult TBAs during pregnancy or for delivery?

………

………

………

………

INTERVIEWER: *Thank you very much for the information and your time!*
